# Distinct Roles for Intracellular and Extracellular Lipids in Hepatitis C Virus Infection

**DOI:** 10.1371/journal.pone.0156996

**Published:** 2016-06-09

**Authors:** Sowmya Narayanan, Albert H. Nieh, Brandon M. Kenwood, Christine A. Davis, Annie-Carole Tosello-Trampont, Tedd D. Elich, Steven D. Breazeale, Eric Ward, Richard J. Anderson, Stephen H. Caldwell, Kyle L. Hoehn, Young S. Hahn

**Affiliations:** 1 Beirne B. Carter Center for Immunology Research, University of Virginia, Charlottesville, United States of America; 2 Department of Microbiology, Immunology, and Cancer Biology, University of Virginia, Charlottesville, United States of America; 3 Department of Pharmacology, University of Virginia, Charlottesville, United States of America; 4 Department of Biology, University of Richmond, Richmond, United States of America; 5 Cropsolution Inc., Morrisville, United States of America; 6 Division of Gastroenterology and Hepatology, University of Virginia, Charlottesville, United States of America; Inserm, U1052, UMR 5286, FRANCE

## Abstract

Hepatitis C is a chronic liver disease that contributes to progressive metabolic dysfunction. Infection of hepatocytes by hepatitis C virus (HCV) results in reprogramming of hepatic and serum lipids. However, the specific contribution of these distinct pools of lipids to HCV infection remains ill defined. In this study, we investigated the role of hepatic lipogenesis in HCV infection by targeting the rate-limiting step in this pathway, which is catalyzed by the acetyl-CoA carboxylase (ACC) enzymes. Using two structurally unrelated ACC inhibitors, we determined that blockade of lipogenesis resulted in reduced viral replication, assembly, and release. Supplementing exogenous lipids to cells treated with ACC inhibitors rescued HCV assembly with no effect on viral replication and release. Intriguingly, loss of viral RNA was not recapitulated at the protein level and addition of 2-bromopalmitate, a competitive inhibitor of protein palmitoylation, mirrored the effects of ACC inhibitors on reduced viral RNA without a concurrent loss in protein expression. These correlative results suggest that newly synthesized lipids may have a role in protein palmitoylation during HCV infection.

## Introduction

The liver is the primary site of synthesis, storage, and oxidation of lipids and other macromolecules. As such, hepatic lipid metabolism is essential for the maintenance of systemic nutrient homeostasis. Dysregulation of hepatic lipid metabolism is a hallmark of several diseases including diabetes, alcoholic and non-alcoholic fatty liver disease, and parasitic and viral infections, including hepatitis C virus (HCV) infection [1–5]. HCV infection is associated with the development of liver disease characterized by chronic hepatic inflammation leading to cirrhosis and hepatocellular carcinoma [6, 7]. At present, as many as 2–3% of the world’s population is infected with HCV, although the recent development of viral protease and polymerase inhibitors has made notable advancements in the treatment of the disease [8–11]. However, many patients are precluded from receiving treatment due to comorbidities or infection with resistant genotypes of HCV [12]. Thus, understanding the pathogenesis of HCV infection is essential for providing insights into the development of novel pan-genotypic therapeutic agents.

The life cycle of HCV relies on hepatic lipids, which results in metabolic disturbances. This manifests clinically as insulin resistance, dysregulated serum lipoproteins, and abnormal accumulation of intracellular lipids, i.e., steatosis [13–19]. These metabolic shifts are partly due to virus-induced increases in *de novo* lipogenesis [17, 20, 21]. Despite the enhanced synthesis of lipids during HCV infection, *de novo* lipogenesis contributes to less than 5% of hepatic lipid stores, indicating that the bulk of lipids available to HCV may be derived from extracellular sources [22–25]. Indeed, the lipoviroparticle, the most infectious form of HCV consisting of virus packaged with triglyceride-rich lipoproteins, harbors a larger fraction of viral RNA post-prandially when compared to fasting states [26–29]. These observations point to an intimate link between HCV and lipids; yet, the specific contributions of *de novo* synthesized lipids compared to those obtained from the extracellular environment have not been well elucidated in HCV infection or other viral diseases. Indeed, considering that changes in host lipid metabolism are characteristic of many positive-strand RNA viruses, understanding the contributions of *de novo* synthesized and exogenous lipids may have significant implications for the biology of formidable pathogens, such as encephalitic Togaviruses and Flaviviruses.

The acetyl-CoA carboxylase enzymes (ACC1 and ACC2) catalyze the rate-limiting step of *de novo* lipogenesis, in which acetyl-CoA is carboxylated to form malonyl-CoA. Malonyl-CoA is subsequently converted to palmitate, a 16-carbon saturated fatty acid. In addition to their role as the building blocks of most lipids, fatty acids also participate in numerous cellular processes, including post-translational modification of proteins. Covalent addition of palmitate to a cysteine residue on proteins, termed S-palmitoylation, regulates protein conformation, stability, function, trafficking to membranes, and interactions with other proteins [30–32]. In addition to the indispensable role of protein palmitoylation in many cellular processes, it also has been reported to play a crucial role in regulating virion composition, infectivity, and evasion of host immune responses [33–35]. In particular, palmitoylation of HCV core and NS4B was previously shown to influence the efficiency of viral assembly and replication [36, 37]. Conversely, while palmitoylation of the host protein CD81 increases susceptibility to HCV, it also confers anti-viral activity to interferon-induced transmembrane (IFITM) proteins [38, 39]. Both exogenously derived and *de novo* synthesized lipids can be used to palmitoylate proteins; however, *de novo* lipogenesis is required for palmitoylation of specific host proteins [32]. Therefore, the metabolic imbalances in *de novo* lipogenesis and extrahepatic lipids in HCV-infected patients may uniquely influence both the virus and the host through changes in protein palmitoylation.

Here, we investigated the respective roles of *de novo* lipogenesis and extracellular lipids in HCV infection using two non-competitive inhibitors of ACC enzymes, K1 and soraphen A. We found that blockade of *de novo* lipogenesis through ACC inhibition decreased HCV RNA by limiting viral replication, lipid droplets available for assembly, and viral export. Supplying ACC inhibitor-treated cells with exogenous fatty acids, the end products of *de novo* lipogenesis, selectively rescued lipid droplets, with no effect on viral replication and release; this suggests that solely repleting lipids is insufficient to overcome the effects of inhibiting *de novo* lipogenesis. Furthermore, inhibiting protein palmitoylation recapitulated the effects of ACC inhibition. These results suggest that intracellular and extracellular lipids contribute differentially to HCV infection.

## Materials and Methods

### Virus, cells, and reagents

The JFH-1 strain of HCV was kindly provided by Takaji Wakita [40]. UV-inactivated virus was generated by exposing virus stocks to 2–3 minutes of UV light in a Stratalinker 3000 (Agilent Technologies). Huh7.5.1 cells were maintained in DMEM with 10% FBS, 100 U/mL penicillin/streptomycin, 2 mM L-glutamine, and 1% non-essential amino acids and infected with cell culture-derived JFH-1 at a multiplicity of infection (MOI) of 0.1. Huh7.5 cells harboring the HCV subgenomic replicon (Huh7.5-SG), a gift from Charles Rice, were cultured in Huh7.5.1 media in the presence of 750 μg/mL of G418 (Invivogen) to maintain viral RNA [41]. K1 and soraphen A were provided by Crop Solution, Inc. K1 was synthesized as described [42]. Sofosbuvir, the NS5B polymerase inhibitor (PSI-7977), was purchased from MedChem Express. The inhibitor of palmitoylation, 2-bromopalmitate (2-BP), was obtained from Sigma-Aldrich.

### Real-time PCR

Cellular RNA was extracted using the GenElute Mammalian Total RNA Miniprep Kit (Sigma-Aldrich) or the RNAeasy Plus Mini Kit (Qiagen). Following reverse transcription with the High Capacity RNA-to-cDNA kit (Life Technologies), RT-PCR was run on the StepOnePlus Real-Time PCR System with Taqman RT-PCR assays (Life Technologies) for ACTB (Assay ID Hs99999903_m1), HPRT1 (Assay ID Hs99999909_m1), and JFH-1 (Custom design; forward 5’-CCTTCACGGAGGCCATGA-3’; reverse 5’-ACAGGATGTTATTAGCTCCAG-GTCATA-3’; probe 5’-CCTCCTGGTGATCCC-3’; FAM reporter; MGB-NFQ quencher). Data are presented as relative fold increases in HCV RNA.

### Crystal violet assay

Huh7.5.1 cells were grown in 96-well plates at 20,000 cells/well. At the time of assessment, culture supernatants were aspirated and cells were treated with crystal violet solution (0.5% crystal violet in 50% methanol/water) for 20 minutes. The stain was solubilized with 1% SDS for 3 hours. Absorbance was read at 570 nm on a PowerWave XS spectrophotometer (BioTek).

### siRNA and plasmid transfections

siRNA pools against ACC1, ACC2, or a negative control were purchased from Dharmacon and transfected with DharmaFECT 4 Transfection reagent. pFR_HCV_xb plasmid, a gift from Phil Sharp (Addgene plasmid #11510), was transfected using Lipofectamine 2000 (Invitrogen) [43]. Luciferase activity for translation assays were determined using the Dual-Luciferase® Reporter Assay System (Promega) and quantified on a GloMax®-Multi Detection System (Promega).

### Mass spectrometry

Cultured cells were trypsinized, centrifuged at 400 × g for 5 minutes at 4°C, and resuspended in PBS. 50 μL of cell lysate was added to 1 mL acidified methanol (0.1 N HCl) containing internal standard cocktails for sphingolipids (containing 0.5 nmol each: C_17_-ceramide, C_12_-glucosylceramide, C_8_-dihydroceramide, and C_12_-sphingomyelin) and glycerolipids (0.1 nmol each of C_15_-diacylglycerol and C_17_-lysophosphatidic acid). Sphingolipids, glycerolipids, and phospholipids were extracted and measured via liquid chromatography-tandem mass spectrometry (LC/MS/MS) as previously described with slight modifications for the measurement of phospholipids [44]. Phosphatidic acids, lysophosphatidic acids, and phosphatidyl-serines were analyzed in negative mode after separation in a Discovery (Supelco) C18 column (50 mm × 2.1 mm, 5 μm bead size). Mobile phase A consisted of 60% acetonitrile, 40% H_2_O, 0.1% formic acid, and 1 mM ammonium acetate. Mobile phase B consisted of 90% isopropyl alcohol, 10% acetonitrile, 0.1% formic acid, and 1 mM ammonium formate. Chromatography was run for a total of 10 min using the following gradient: 1 minute 100% solvent A; a linear gradient to 100% solvent B over 6 min; 2 min 100% solvent B; 1 min 100% solvent A. Total flow was 0.6 ml/min. Total values were normalized to protein concentration. Significance in fold change was determined by Student’s *t* test (*p*<0.05) and further corrected by a false discovery rate adjustment (*p*<0.1).

### Immunoblot analysis

Cells were lysed in radioimmunoprecipitation assay (RIPA) lysis buffer, suspended in Laemmli buffer, resolved on a 4–15% Mini-PROTEAN® TGX gel (Biorad), and blotted on a PVDF membrane with antibodies for ACC1 (Millipore), ACC2 (Cell Signaling), HCV core (Anogen), NS3 (Abcam), and vinculin (Cell Signaling Technology).

### HCV titration

Virus stocks generated from culture supernatants of Huh7.5.1 cells following 6–8 days of infection were centrifuged to remove debris. HCV titer was determined by infecting Huh7.5.1 cells in Lab-Tek® chamber slides at various dilutions for 3 days, after which the cells were fixed in 4% paraformaldehyde/PBS. Cells were blocked in 0.3% Triton-X/5% goat serum, stained using mouse anti-HCV core antigen antibody (Thermo Scientific) and highly cross-adsorbed APC goat-anti-mouse IgG (Life Technologies), and mounted in ProLong® Gold Antifade Mountant with DAPI (Life Technologies). Images were captured on Zeiss LSM 710 Multiphoton microscope and the number of focus forming units was calculated from at least 10 fields in each experiment.

### Confocal microscopy

Cells were stained as described under “HCV titration.” For assembly studies, bodipy (Life Technologies) was added along with the secondary antibody. Adjustments to brightness and contrast made in Adobe Photoshop CS were kept to a minimum and applied to all images from a given experiment. The number of pixels was quantified over 10 fields/condition in each experiment using ImageJ. In brief, each image was split into individual channels. Thresholds for each channel were kept constant within each experiment. The “measure” function was used to quantify the number of pixels in each channel. To calculate the percentage of red pixels that co-localized with green pixels, both channels were inverted, after which a selection was created for red pixels and pasted onto the green channel using the “ROI (region of interest) manager” tool.

### Addition of exogenous fatty acids

Sodium salts of palmitate, oleate, and linoleate were purchased from Sigma-Aldrich and mixed in methanol in a 1:2:1 ratio [45]. The methanol was removed using nitrogen and the fatty acid mixture was reconstituted in culture media containing 0.25% fatty acid free BSA (Sigma-Aldrich) to a final concentration of 25 μM palmitate, 50 μM oleate, and 25 1M linoleate.

### Protein aggregate staining

Huh7.5.1 cells were grown in Lab-Tek® chamber slides. Protein aggregates were stained using the ProteoStat® Aggresome detection kit (Enzo Life Sciences) as per the manufacturer’s instructions. Images were captured on Zeiss LSM 710 Multiphoton microscope with 5–10 fields for each condition per experiment.

### Statistical analysis

Results are the mean ± SEM. Statistical significance was determined by an unpaired *t* test, one-way analysis of variance (ANOVA) with Tukey’s post-test, or two-way ANOVA with Bonferroni post-test. Statistical analyses were performed using Prism GraphPad software v4.0c. ns: not significant, *p<0.05, **p<0.01, ***p<0.001.

## Results

### ACC inhibition decreases intracellular HCV RNA

**Fig 1:**
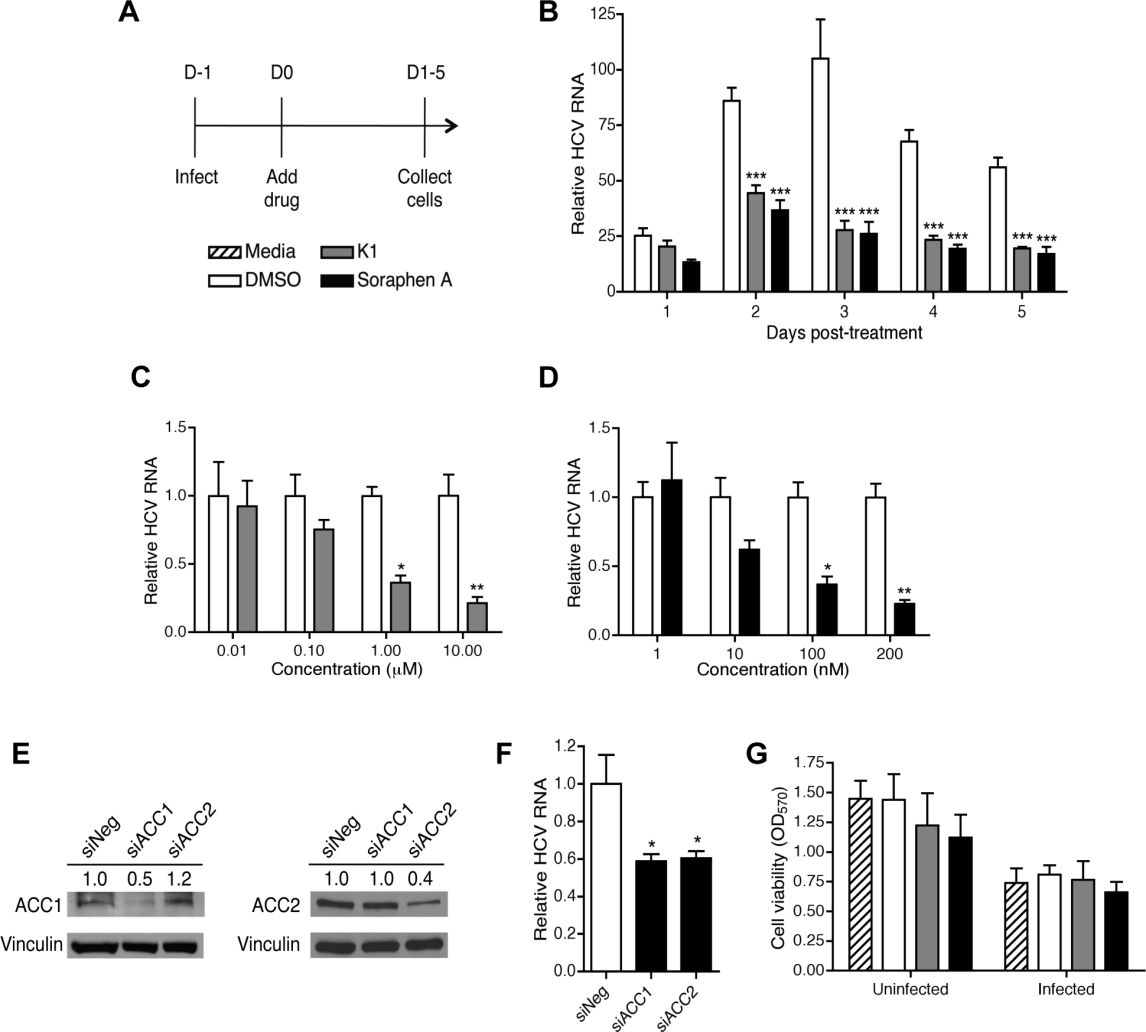
Inhibition of *de novo* lipogenesis decreases intracellular HCV RNA. (A) Experimental setup. (B) Kinetics of changes in HCV RNA. Infected Huh7.5.1 cells were treated with 1 μM K1 or 100 nM soraphen A. DMSO was added at an equivalent volume. The media was replaced with fresh ACC inhibitors on D3 post-treatment (PT). HCV RNA was detected by qRT-PCR. The plotted values are fold increases relative to D0 PT (1 day post-infection, before addition of ACC inhibitors). (C, D) Dose response of K1 (C) and soraphen A (D). Infected Huh7.5.1 cells were treated with DMSO, K1, or soraphen A for 3 days before qRT-PCR analysis. (E, F) Silencing of ACC1 and ACC2. Infected Huh7.5.1 cells were transfected with a pool of siRNA against ACC1 or ACC2 for 3 days before immunoblot (E) and qRT-PCR analysis (F). (G) Cell viability upon treatment with K1 and soraphen A. Uninfected and infected Huh7.5.1 were treated with media, DMSO, 1 μM K1, or 100 nM soraphen A for 3 days. Viability was determined by crystal violet staining. Results are the representative or mean ± SEM of 3–5 independent experiments. Statistical significance was calculated by two-way ANOVA with Bonferroni post-tests (B-D) or one-way ANOVA with Tukey’s post-test (F). nd: not detected, *p<0.05, **p<0.01, ***p<0.001.

*De novo* lipogenesis, the process of generating fatty acids from acetyl-CoA, is upregulated upon expression of HCV proteins [20, 46, 47]; however, the specific contribution of enhanced *de novo* lipogenesis to HCV infection is not well defined. The enzymes ACC1 and ACC2 catalyze the rate-limiting step of *de novo* lipogenesis and represent a potential pharmacological target for delineating the function of *de novo* synthesized lipids in HCV infection. Utilizing a well-established *in vitro* model of HCV infection, i. e., the human hepatocyte cell line Huh7.5.1 infected with the JFH-1 strain of HCV, we treated hepatocytes at D1 post-infection with two non-competitive ACC inhibitors, K1 or soraphen A, or vehicle control (DMSO) (Fig 1A; see also S1A and S1B Fig) [48]. While intracellular HCV RNA was notably decreased in K1 and soraphen A-treated cells compared to vehicle-treated cells beginning at D2 post-treatment (PT), this decrease was most prominent at D3 PT, and was maintained through D5 PT (Fig 1B). The effect on viral RNA was dose-dependent for both K1 and soraphen A, and began to plateau at doses of 1 μM K1 and 100 nM soraphen A (Fig 1C and 1D). All subsequent experiments were therefore conducted at D3 PT with 1 μM K1 and 100 nM soraphen A.

Next, we confirmed that the effect on viral RNA was due to specific inhibition of ACC through transient knockdown of the two ACC isoforms—ACC1 and ACC2. Viral RNA was decreased even with a partial loss of ACC1 or ACC2, verifying that inhibition of *de novo* lipogenesis leads to a loss in intracellular HCV RNA (Fig 1E and 1F). Importantly, inhibition of *de novo* lipogenesis did not significantly affect the viability of uninfected or infected cells, as total DNA content, redox capacity, and ATP production were similar among all treatment groups (Fig 1G; see also S2 Fig). These results indicate that inhibition of *de novo* lipogenesis via K1 and soraphen A treatment significantly decreases intracellular HCV RNA without compromising hepatocyte viability.

### ACC inhibition limits HCV replication, lipid droplets required for viral assembly, and virion production

**Fig 2:**
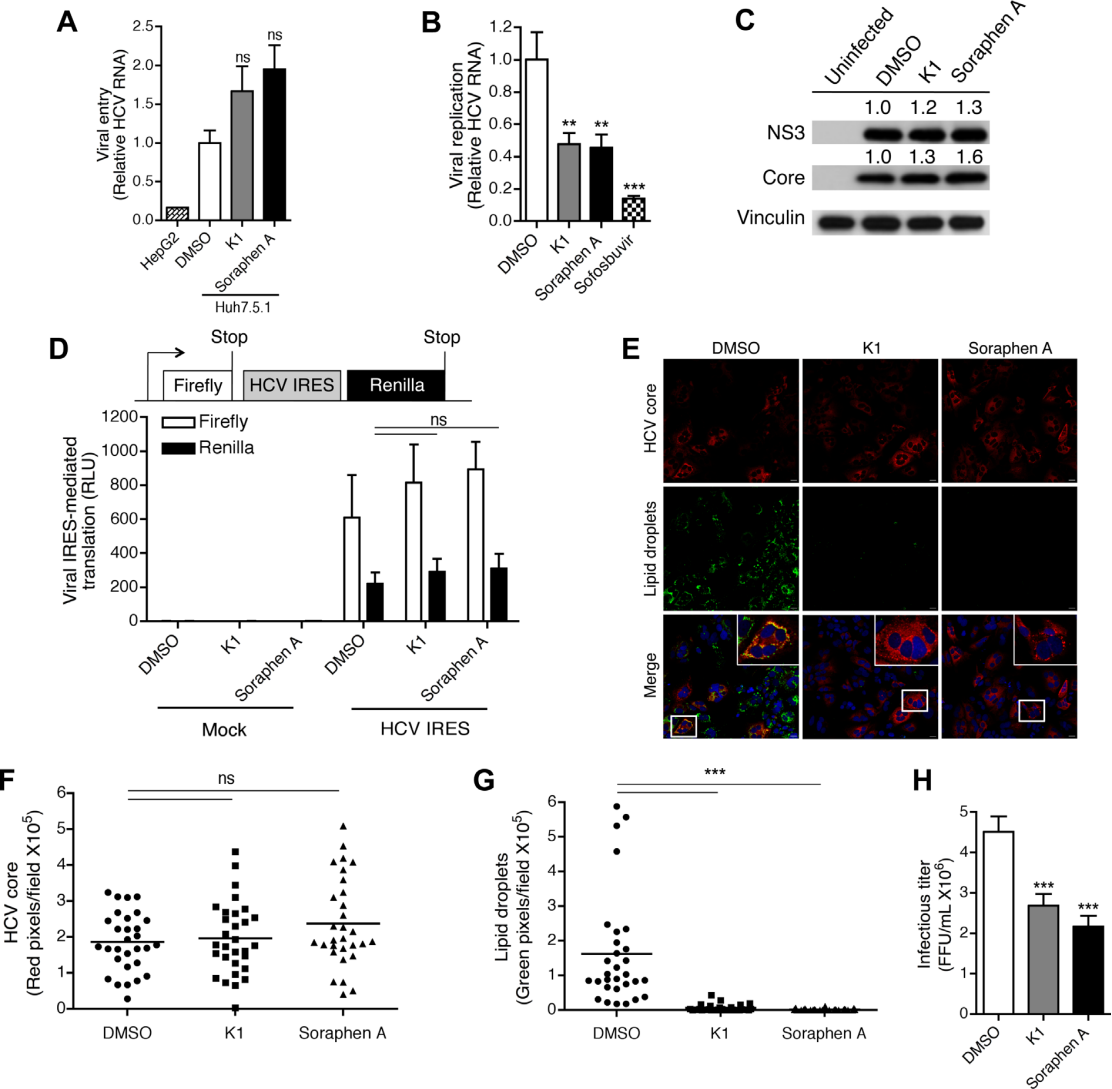
*De novo* lipogenesis is required for HCV replication and release and supplies cellular lipid droplets that may contribute to viral assembly. (A) Effect on entry. Huh7.5.1 cells were treated with DMSO, 1 μM K1, or 100 nM soraphen A for 3 days after which the media was replaced with JFH-1 (MOI 0.1). HepG2 cells were used as a negative control. Cells were collected for qRT-PCR analysis at 1 hour post-infection. (B) Effect on replication. Huh7.5 cells harboring HCV subgenomic replicons were treated with DMSO, 1 μM K1, 100 nM soraphen A, or 250 nM of the NS5B polymerase inhibitor, sofosbuvir, for 3 days. HCV RNA was detected by qRT-PCR. (C) Effect on viral protein. Infected Huh7.5.1 cells were treated with DMSO, 1 μM K1, or 100 nM soraphen A for 3 days. Intracellular viral protein was assessed by immunoblotting. Densities of NS3 and core staining were calculated relative to vinculin and then normalized to DMSO. (D) Effect on translation. Huh7.5.1 cells were transfected with the bicistronic pFR_HCV_xb construct in which the HCV IRES regulated translation of renilla luciferase. Mock transfected cells served as controls. Twenty-four hours post-transfection, the media was replaced with DMSO, 1 μM K1, or 100 nM soraphen A for 3 days. Cellular lysates were assessed for both firefly and renilla luciferase activity. (E-G) Effect on assembly. Infected Huh7.5.1 cells were treated with DMSO, 1 μM K1 or 100 nM soraphen A for 3 days and stained for the nucleocapsid core protein and lipid droplets. Number of red (HCV core, F) and green (lipid droplets, G) was quantified over 10 fields in each experiment. Scale bar is equivalent to 20 μm. (H) Effect on infectious titer. Huh7.5.1 were infected with serially diluted supernatants of infected Huh7.5.1 cells that had been treated with DMSO, 1 μM K1, or 100 nM soraphen A for 3 days. The number of HCV-core positive focus forming units (FFU) was quantified 3 days post-infection. Results are the representative or mean ± SEM of 3–6 independent experiments. Statistical significance was calculated by one-way ANOVA with Tukey’s post-test. ns: not significant, **p<0.01, ***p<0.001.

The decrease in HCV RNA observed upon ACC inhibition may reflect changes in one or more steps of the HCV life cycle. Therefore, we sought to identify the effects of ACC inhibition on multiple steps of the HCV life cycle, namely entry, replication, translation, assembly, and release of infectious virions. We first examined the effects of K1 and soraphen A on viral entry. Negative-strand HCV RNA, which is an RNA intermediate during viral replication, appears within five hours of infection *in vitro* [49]. In order to exclude any replicated viral RNA intermediates in our analysis, we measured the genomic viral RNA in K1 and soraphen A-treated Huh7.5.1 cells infected with HCV for one hour. Untreated HepG2 cells, which do not express CD81 and are thus less permissive to infection with JFH-1, were used as a negative control. Cells treated with either K1 or soraphen A had equivalent, if not increased, intracellular viral RNA compared to vehicle-treated cells (Fig 2A), suggesting that a defect in viral entry was not contributing to the loss in intracellular HCV RNA following inhibition of *de novo* lipogenesis. We next evaluated the effect of ACC inhibition on viral replication using Huh7.5-SG cells, which harbor an HCV subgenomic replicon lacking structural proteins. These cells do not produce intact virus capable of initiating secondary infections, yet provide an ideal model to study the impact of inhibiting *de novo* lipogenesis on HCV replication. Similar to treatment of Huh7.5.1 cells infected with infectious virus, addition of either K1 or soraphen A significantly reduced viral RNA (Fig 2B). These findings were further supported by a similar loss in viral RNA in cells treated with the polymerase inhibitor, sofosbuvir. *De novo* lipogenesis thus plays a critical role in viral RNA synthesis.

Given that the inhibition of *de novo* lipogenesis decreased replication of viral RNA, we expected to see a similar loss in viral proteins. Surprisingly, the amounts of HCV core and NS3 proteins were comparable between DMSO, K1, and soraphen A-treated cells (Fig 2C). To address if the mismatch in viral RNA and protein was due to an increase in translation, we transfected cells with a bicistronic construct expressing renilla luciferase under the control of the HCV internal ribosome entry site (IRES) [43]. Firefly luciferase activity is thus an indicator of transfection efficiency while renilla luciferase activity is a measure of translation directed by the HCV IRES. As seen in Fig 2D, both firefly and renilla luciferase activities were comparable among all three treatment groups. These results suggest that the discrepancy between viral RNA and viral protein levels resulting from the inhibition of ACC was not due to enhanced translation through the HCV IRES.

We next evaluated subsequent steps of the viral life cycle. To this end, we tested the effect of ACC inhibitors on viral assembly. HCV assembly requires the co-localization of the HCV core protein with lipid droplets [50–52]. As expected, DMSO-treated cells had an abundance of core protein that co-localized with lipid droplets (Fig 2E–2G). In contrast, K1 and soraphen A-treated cells displayed a marked loss of lipid droplets, yet retained expression of HCV core as demonstrated in the immunoblots of total lysates (Fig 2C, 2E and 2F; see also S3 Fig). The prerequisite for viral assembly, namely, the colocalization of HCV core and lipid droplets, thus appeared to be compromised in cells with reduced ACC activity. These findings indicate that *de novo* lipogenesis plays a critical role in propagating HCV in part by supplying the scaffold for viral assembly. Lastly, we assessed viral titer in the supernatants of cells treated with ACC inhibitors and found that the infectious titer was reduced in K1 and soraphen A-treated cells when compared to the vehicle controls (Fig 2H). Collectively, these results demonstrate that ACC inhibition affects multiple steps of the HCV life cycle, specifically replication, assembly, and production of infectious virions.

**Fig 3:**
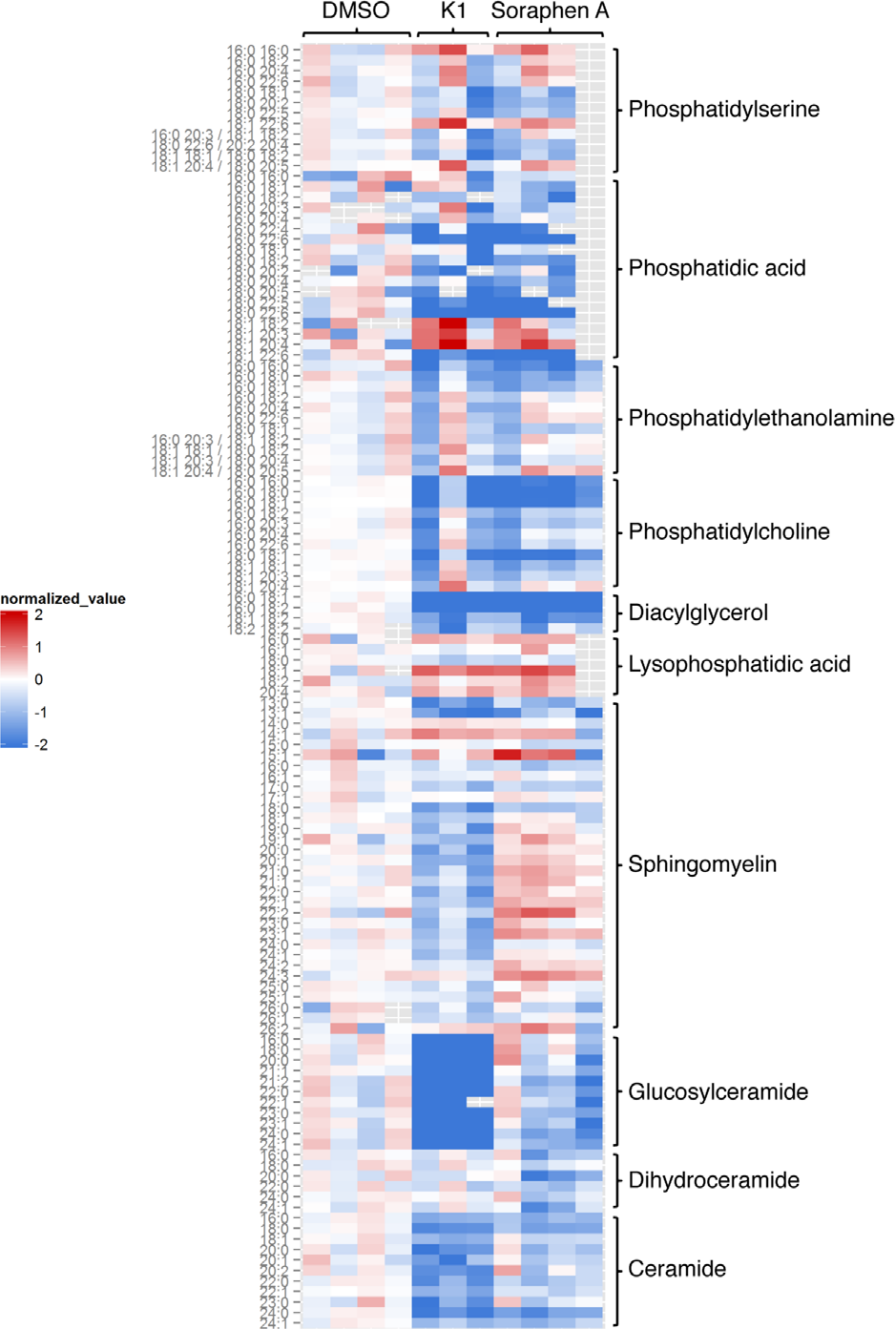
Inhibiting *de novo* lipogenesis leads to broad changes in the hepatocyte lipidome. Infected Huh7.5.1 cells were treated with DMSO, 1 μM K1, or 100 nM soraphen A for 3 days. Indicated lipids were quantified by mass spectrometry and are plotted as normalized values relative to the average in DMSO treated cells. Fatty acid chain length and degree of saturation are indicated on the left. Hashed gray boxes represent replicates that were not detected by the spectrometer. Results are from 3–4 independent experiments.

Given the loss of lipid droplets in K1 and soraphen A-treated cells, we sought to identify changes in the lipidome that may be contributing to the loss of viral replication, assembly, and virion production. We used liquid chromatography tandem mass spectrometry (LC-ESI-MS/MS) to measure different lipid species in infected hepatocytes treated with DMSO, K1, or soraphen A. As expected, the majority of lipids measured were decreased upon inhibition of ACC, particularly phosphatidic acids, phosphatidylcholines, diacylglycerols, and ceramides (Fig 3; see also S1 Table). As these lipids are key players in maintenance of cellular membranes, their loss upon treatment with K1 and soraphen A may be indicative of changes in the quality of intracellular membranes that facilitate viral replication.

### Exogenous fatty acids restore lipid droplets but fail to rescue viral replication and virion production

**Fig 4:**
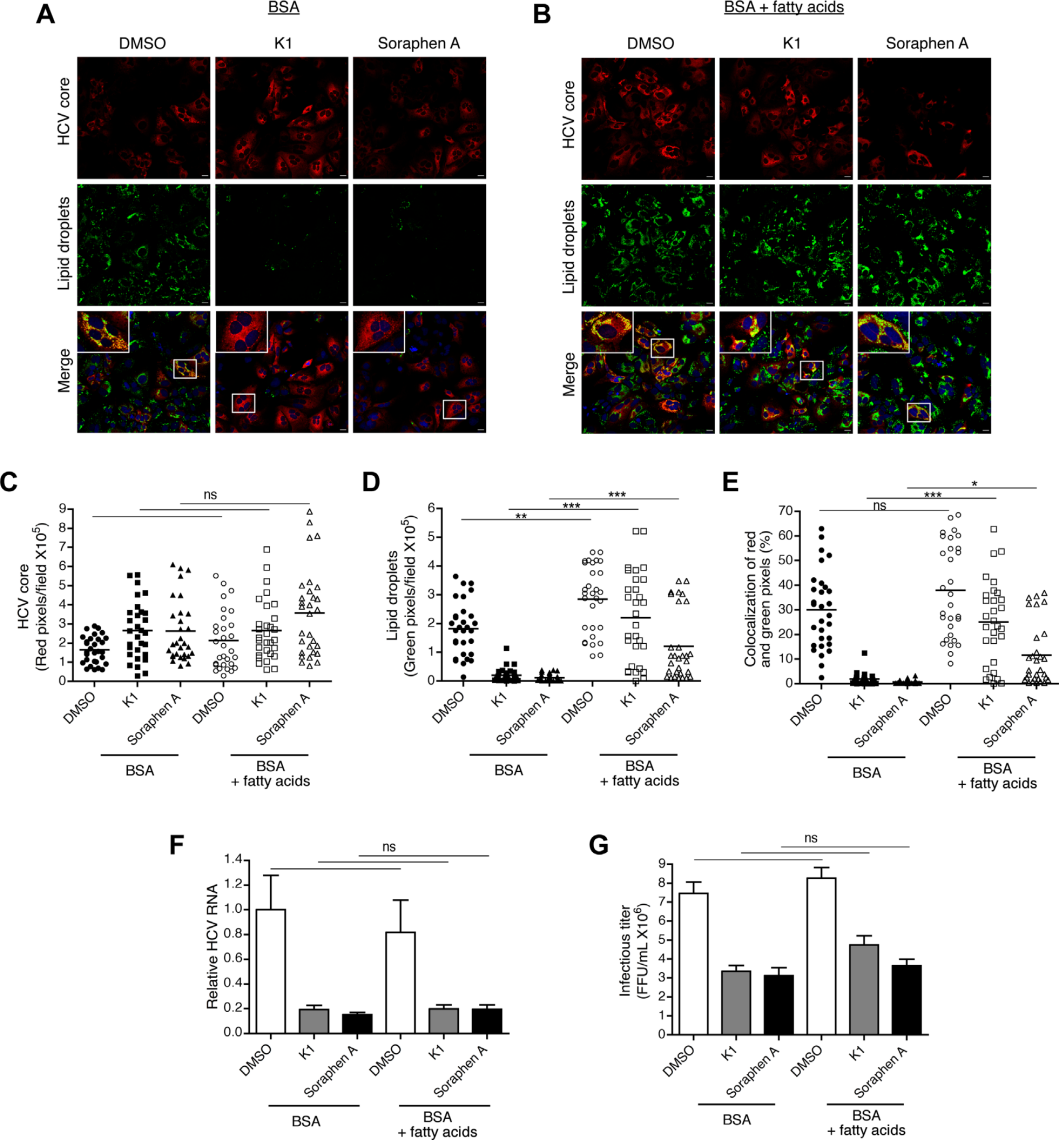
Exogenous lipids contribute to HCV assembly via lipid droplet formation but are dispensable for replication and release. (A-E) Infected Huh7.5.1 cells were treated with 1 μM K1, 100 nM soraphen A, or an equivalent volume of DMSO in media containing BSA (A) only or BSA + fatty acids (palmitate, oleate, and linoleate) (B). At D3 of treatment, cells were stained for the HCV nucleocapsid core protein and lipid droplets. Nuclei are indicated in blue. Areas of co-localization may be indicative of assembly of infectious virions. Scale bar is equivalent to 20 μm. Number of red (HCV core, C) pixels and green (lipid droplets, D) pixels were quantified over 10 fields in each experiment. Percentage of red pixels that colocalized with green pixels (E). (F) Infected Huh7.5.1 cells were treated as described in A-E. Intracellular HCV RNA was detected by qRT-PCR. (G) Supernatants of cells treated as described in A-E were used to infect Huh7.5.1 cells for 3 days after which the number of core positive foci was quantified. Results are the representative or mean ± SEM of 3–5 independent experiments. Statistical significance was calculated by one-way ANOVA with Tukey’s post-test. ns: not significant, *p<0.05, **p<0.01, ***p<0.001.

The end product of *de novo* lipogenesis is palmitate, a 16-carbon saturated fatty acid, which can then be modified to generate a diverse repertoire of lipids. Given that inhibition of *de novo* lipogenesis decreases HCV replication, cellular lipid droplets, and infectious titer, we tested whether supplementing fatty acids would rescue these defects. We added a mixture of saturated and unsaturated fatty acids including palmitate, oleate, and linoleate, at concentrations found in blood, to cells treated with ACC inhibitors [45]. Notably, cells were treated with ACC inhibitors in media containing serum; as such, the addition of these fatty acids is in excess of fatty acids in the serum. Supplementing with the fatty acid mixture restored lipid droplets in K1 and soraphen A-treated cells (Fig 4A, 4B and 4D). Importantly, the lipid droplets co-localized with HCV core, indicating that extracellular sources of fatty acids contribute to lipid droplet formation even in the absence of *de novo* lipogenesis (Fig 4A–4E). However, supplementation with fatty acids did not restore intracellular HCV RNA or the infectious virions released in culture supernatants (Fig 4F and 4G). These results indicate that viral replication is a limiting factor for the production of infectious virus. In addition, these results support the hypothesis that while *de novo* lipogenesis is necessary for viral replication, exogenous sources of lipids can supply triglycerides for the formation of lipid droplets that contribute to HCV assembly.

### Inhibiting protein palmitoylation mimics effects of ACC inhibition

**Fig 5:**
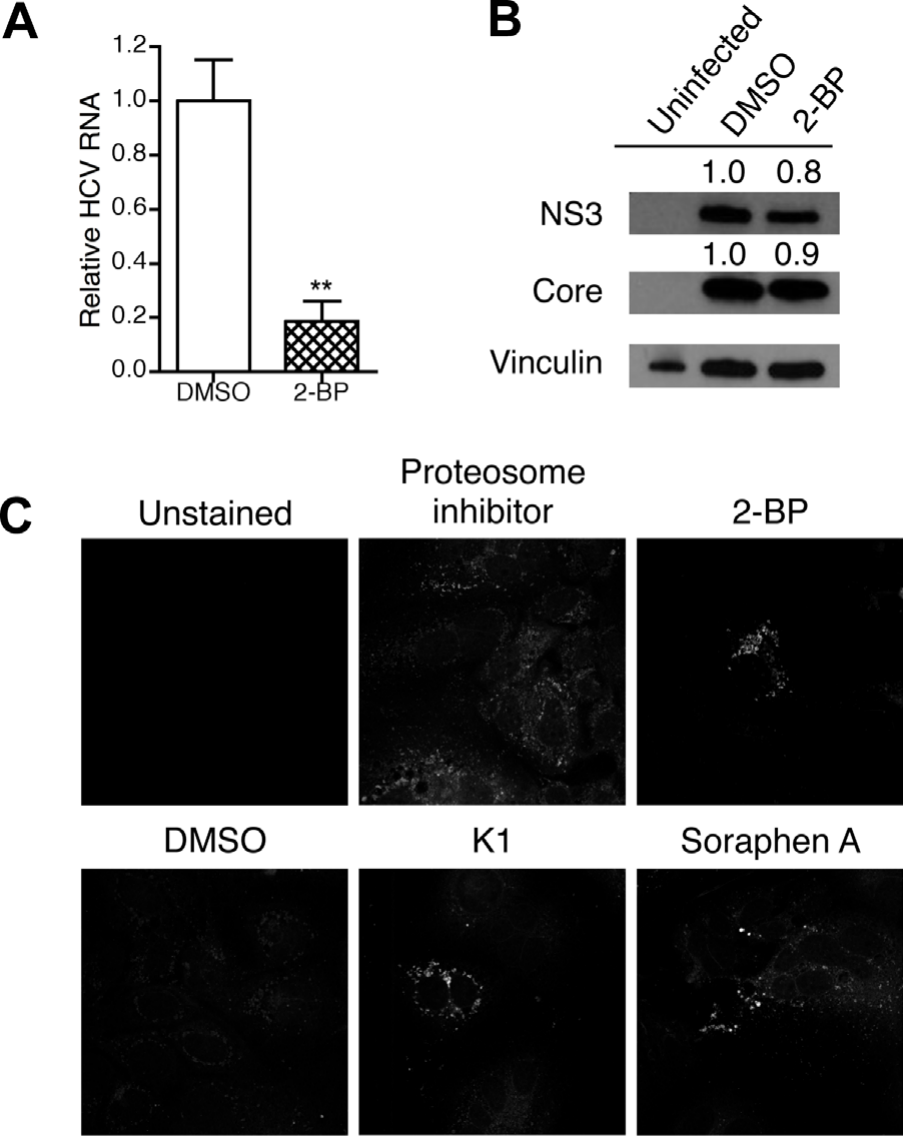
Inhibition of protein palmitoylation also leads to a loss in viral RNA without a concurrent loss in viral protein. (A, B) Infected Huh7.5.1 cells were treated with 60 μM 2-bromopalmitate (2-BP) or an equivalent volume of DMSO for 3 days. A, Intracellular HCV RNA was detected by qRT-PCR. B, Intracellular viral protein was assessed by immunoblotting. Densities of NS3 and core were calculated relative to vinculin and then normalized to DMSO. (C) Infected Huh7.5.1 cells were treated with DMSO, 1 μM K1, 100 nM soraphen A, or 60 μM 2-BP for 3 days and stained for protein aggregates. Cells treated for 6 hours with 5 μM MG-132, a proteosome inhibitor, served as the positive control. Results are the representative or mean ± SEM of 3–4 independent experiments. Statistical significance was calculated by an unpaired *t* test. **p<0.01.

Fatty acids are essential for several cellular functions including post-translational modification of proteins by palmitoylation. Palmitoylation of HCV core and NS4B was previously shown to be required for optimal production of virions and formation of the HCV replication complex, respectively [36, 37]. We therefore investigated whether inhibiting protein palmitoylation during HCV infection would produce results similar to inhibition of *de novo* lipogenesis. To test this hypothesis, HCV-infected cells were treated with 2-bromopalmitate (2-BP), a competitive inhibitor of palmitoyl acyltransferases. Our results demonstrate that addition of 2-BP to HCV-infected hepatocytes decreased viral RNA with minimal loss in viral protein, recapitulating the effects of ACC inhibitors (Fig 5A and 5B). Depalmitoylation of proteins is known to alter their cellular membrane localization, function, and aggregate formation [53, 54]. Previous studies had demonstrated that palmitoylation of HCV core regulated trafficking to ER membranes, while palmitoylation of NS4B was necessary for its interaction with other viral proteins [36, 37]. In addition to these well-established functions of palmitoylation in HCV infection, our study investigated the function of this process in protein aggregate formation. As seen in Fig 5C, protein aggregates were increased in cells treated with K1, soraphen A, or 2-BP compared to those treated with DMSO. Cells treated with a proteasome inhibitor served as the positive control. These results may be indicative of mislocalization of viral proteins, essential host factors, or both. In fact, as the requirement for palmitoylation of NS4B in HCV replication was recently challenged, our results may be more suggestive of defects in palmitoylation of host proteins [55]. Nonetheless, although the similarities with 2-BP treatment are correlative and do not definitely prove a role for ACC inhibitors in regulating protein palmitoylation, they offer the possibility that *de novo* lipogenesis may facilitate palmitoylation of proteins necessary for optimal replication of HCV.

## Discussion

The link between lipid metabolism and HCV is a well-defined relationship that is thought to partly dictate the tropism of the virus to the liver [56, 57]. HCV-induced upregulation of *de novo* lipogenesis contributes to viral replication, assembly, and packaging for export through biogenesis of membranes, lipid droplets, and lipoproteins. In this report, we define an additional role for *de novo* lipogenesis in HCV infection where the specific inhibition of ACC activity, which catalyzes the rate-limiting step of *de novo* lipogenesis, decreases viral RNA replication without a concurrent loss in viral protein levels. Importantly, inhibition of protein palmitoylation in the infected host mirrored the effects of inhibiting the ACC enzymes, suggesting a potential role for ACC inhibitors in altering HCV replication through palmitoylation. Moreover, ACC inhibition resulted in a notable reduction in lipid droplets, which were restored by the addition of exogenous fatty acids. Collectively, our results posit distinct roles for *de novo* synthesized and extracellular lipids in HCV infection: *de novo* lipogenesis facilitates viral RNA replication potentially through palmitoylation of host and viral proteins, while exogenous lipids are trafficked to lipid droplets that act as scaffolds for viral assembly.

To dissect the roles of intracellular and extracellular pools of lipids in HCV infection, we used K1 and soraphen A, two non-competitive inhibitors of ACC, the enzyme that catalyzes the rate-limiting step of *de novo* lipogenesis. ACC is a large multi-domain enzyme that exists in two isoforms, ACC1 and ACC2, both of which are expressed in the liver [58]. ACC1 is localized to the cytosol where it functions in carboxylating acetyl-CoA to malonyl-CoA for nascent fatty acid synthesis. ACC2 is targeted to the mitochondria where it inhibits β-oxidation of fatty acids. Although ACC1 is thought to be responsible for the bulk of fatty acid synthesis, previous studies have demonstrated that ACC2 can also function in *de novo* lipogenesis [59]. It is likely for this reason that even partial inhibition of either ACC isoform resulted in a loss in viral RNA.

Our finding that inhibition of ACC decreases viral replication corroborates previous studies demonstrating that lipogenesis is essential for HCV replication. Specifically, previous reports identify a role for fatty acids in the formation of the membranous web, which is the site of HCV replication [60–62]. Our findings do not exclude this possibility as lipidomic analysis revealed a significant loss in lipids in cells treated with K1 or soraphen A for 3 days (Fig 3 and S1 Table). Surprisingly, treatment with K1 resulted in a loss of sphingomyelins in contrast to the slight increase in these lipids upon soraphen A treatment. In addition, K1 treatment produced a more pronounced loss in ceramides and glucosylceramides, with little to no effect on dihydroceramides, when compared to the effects of soraphen A. We cannot discount the possibility that these differences are due to off-target effects of the drug, which may also be contributing to the effect on HCV. Nevertheless, the concerted loss of other classes of lipids in both K1 and soraphen A-treated cells suggests that specific inhibition of ACC may be a common mechanism by which these inhibitors impact HCV infection. For example, the loss of diacylglycerols substantiated the absence of lipid droplets upon ACC inhibition, since diacylglycerols are the precursor to triacylglycerols, the predominant lipid found in lipid droplets. Notably, the striking loss of phosphatidic acids and phosphatidylcholines indicated potential changes to cellular membranes, as both classes of lipids are major membrane components [63]. Therefore, in keeping with previous studies, altered cellular membranes are thus likely contributing to the loss in viral RNA seen upon inhibition of *de novo* lipogenesis [62]. In addition, viral RNA in membranous webs has a longer half-life than its cytosolic counterpart [49], providing a potential explanation for the loss in viral RNA in K1 and soraphen A-treated cells.

Our findings also suggest that HCV assembly may be compromised in K1 and soraphen A-treated cells as lipid droplets were notably lost upon ACC inhibition. Previously, the association of HCV core with the surface of lipid droplets was thought to facilitate viral assembly as disrupting this association significantly reduced virus production [50, 51]. In recent years, this view has been challenged by reports of the ER being the more critical site for HCV assembly [64, 65]. It is therefore possible that the loss of lipid droplets in K1 and soraphen A-treated cells does not necessarily indicate a defect in viral assembly as core could contribute to assembly at the ER instead. However, these reports delineating the role of the ER in HCV assembly employed genomes of HCV isolates other than JFH-1 or assessed JFH-1 after an extended period of culture [64, 65]. Our results may thus represent the impact of ACC inhibitors on HCV assembly exclusively in JFH-1 isolates early in infection. Future investigations that evaluate the effect of ACC inhibitors on other HCV genotypes may help identify a more universal function of *de novo* lipogenesis in HCV assembly.

Nonetheless, to our knowledge, this study is the one of the few reports in which viral protein does not parallel viral RNA in HCV infection. Differences in experimental systems could explain this discrepancy as previous studies investigating the role of *de novo* lipogenesis targeted pathways downstream of ACC, employed transfected HCV replicons or chimeric viruses, or infected cells with cell-culture derived HCV for longer than 24 hours before adding lipid-depleting agents [60–62, 66–68]. In contrast, our studies were performed by inhibiting *de novo* lipogenesis at 24-hours post-infection with cell-culture derived HCV. As HCV RNA is exponentially increased early in infection and begins to plateau at 30–72 hours post-infection [49], the time at which inhibition of *de novo* lipogenesis is initiated may be important. Therefore, the discrepancy in viral RNA and protein levels may reflect the early impact of *de novo* lipogenesis in HCV infection, which is compounded over time with changes in membrane lipids, and distinct from the contributions of exogenous lipids.

In particular, protein palmitoylation may be one of the events dependent on *de novo* lipogenesis early in infection. More specifically, palmitoylation of host or viral proteins necessary for HCV replication may be temporally or spatially coupled to *de novo* lipogenesis, such that the target proteins would not be palmitoylated upon the addition of ACC inhibitors. As a result, these host or viral factors would not be able to participate in viral replication, yet would continue to be translated from input viral RNA and the low levels of replicated RNA. The fates of these depalmitoylated proteins could be explained by the increased incidence of protein aggregates upon treatment of HCV-infected hepatocytes with ACC inhibitors or 2-bromopalmitate. Indeed, previous studies have established that loss of palmitoylation does not necessarily target the protein for degradation; instead, depalmitoylated proteins accumulate as stable aggregates [30, 69, 70]. The diversion of proteins to these aggregates may be one explanation for the accumulation of viral protein in cells treated with ACC inhibitors. These data thus raise the possibility that inhibition of *de novo* lipogenesis results in redirection of depalmitoylated host and, potentially, viral proteins to aggregates where they cannot function in replicating the viral genome. However, addition of a mixture of exogenous fatty acids restored lipid droplets without rescuing viral replication or infectious titer. We therefore hypothesize that palmitoylation of proteins that participate in HCV replication may require *de novo* lipogenesis and be independent of exogenously derived lipids.

Alternatively, ACC inhibition could result in the deliberate diversion of exogenously derived fatty acids, or fatty acids liberated from intracellular sources, away from proteins that require palmitoylation. This phenomenon of actively directing fatty acids toward various metabolic fates is known as channeling [71]. It is intriguing to speculate that inhibition of *de novo* lipogenesis during viral infection forces the cell to channel the limited supply of fatty acids towards alternative fates. Such tactics may help combat invasion by the virus by sequestering metabolites essential for viral propagation. These findings may be particularly relevant to infections with HCV genotype 3, which is characterized by extensive steatosis that corresponds to the course of infection [72, 73]. Selective depletion of hepatic lipids in genotype 3 infections may be a promising alternative to direct acting anti-viral agents, especially since these treatments are ineffective against this genotype [8, 74]. Moreover, as steatosis can initiate and exacerbate chronic hepatic inflammation, hypolipidemic agents like the ACC inhibitors may help reduce tissue damage caused by persistent immune responses.

Even before HCV was identified as the causative agent of hepatitis C, its link with hepatic lipid metabolism was foreshadowed by the high incidence of steatosis in patients with what was then called non-A, non-B hepatitis [75]. Our results establish a putative distinction between *de novo* synthesized and exogenously derived lipids in HCV infection using a novel ACC inhibitor, K1, in comparison to an established counterpart, soraphen A. Our findings have important implications for all Flaviruses and other positive-sense RNA viruses, which rely extensively on manipulating host lipid metabolism for their propagation [76–79]. Future studies examining whether host-derived metabolites in turn dictate the metabolome of invading microbes may help identify novel points of therapeutic intervention. In addition, further investigation of the relationship between cellular metabolic processes and pathogens will help improve our understanding of the selective pressures driving the evolution of host-microbe interactions.

## Supporting Information

S1 Fig Role of ACC in *de novo* lipogenesis. (A) ACC catalyzes the rate-limiting step of fatty acid synthesis by converting acetyl-CoA to malonyl-CoA. (B) Structure of novel ACC inhibitor, K1.
(TIFF)

S2 Fig Inhibition of *de novo* lipogenesis does not significantly alter cell viability. (A) Uninfected and infected Huh7.5.1 were grown in 96-well plates at 20,000 cells/well and treated with media, DMSO, 1 μM K1, or 100 nM soraphen A for 3 days. Effect on cell viability was determined by the MTT assay. Briefly, three hours before reading the absorbance, the cells were incubated at 37°C with 20 μL/well of 5 mg/mL of MTT (3-(4,5-dimethylthizaol-2-yl)-2,5-diphenyltetrazolium bromide). Precipitates were solubilized in isopropanol containing 4 mM HCl and 0.1% NP-40. Absorbance was read at 570 nm on a BioTek PowerWave XS. (B) Cells were grown as described in (A). Intracellular ATP content was quantified using the CellTiter-Glo® Luminescent Cell Viability Assay (Promega) according to the manufacturer’s instructions. Results are the mean ± SEM of 3 independent experiments.
(TIFF)

S3 Fig Inhibition of *de novo* lipogenesis leads to a loss of lipid droplets. Infected Huh7.5.1 cells were treated with DMSO, K1, and Soraphen A for 3 days. Cell cultures were fixed in 4% paraformaldehyde/2.5% glutaraldehyde in PBS, post-fixed with 1% osmium tetroxide and potassium ferricyanide, dehydrated in ethanol, and embedded in Epon 812. Sections were cut on a Leica Ultracut UCT at a thickness of 60–80 nm and placed on 200 mesh copper grids for viewing in a JEOL 1010 transmission electron microscope. Images were obtained with a Hamamatsu ORCA-HR. Lipid droplets are indicated by the arrowheads. Scale bar is equivalent to 2 μm. Images are representative of 2–3 independent experiments.
(TIFF)

S1 Table Lipidomics of HCV-infected hepatocytes treated with ACC inhibitors. Infected Huh7.5.1 cells were treated with DMSO, 1 μM K1, or 100 nM soraphen A for 3 days. Indicated lipids were quantified by liquid chromatography tandem mass spectrometry (LC-ESI-MS/MS) and mean values were used to determine significant changes in K1 and soraphen A-treated cells compared to DMSO control. The column “*p* value” was determined by student’s *t* test and *p*<0.05 (bolded) was considered significant. The column “corrected *p* value” was adjusted for false discovery rate (FDR). Because FDR is more stringent than a *t* test, *p*<0.1 (bolded) was considered significant. Positive and negative values in the column “Log (fold change)” indicate an increase or decrease in the lipid, respectively. Results are the mean ± SEM of 3–4 independent experiments.
(PDF)
