# Supplementary figures and images for: Distinct Roles for Intracellular and Extracellular Lipids in Hepatitis C Virus Infection
Source: PLoS One. 2016 Jun 9;11(6):e0156996. doi: 10.1371/journal.pone.0156996 (PMC4900644; doi:10.1371/journal.pone.0156996)

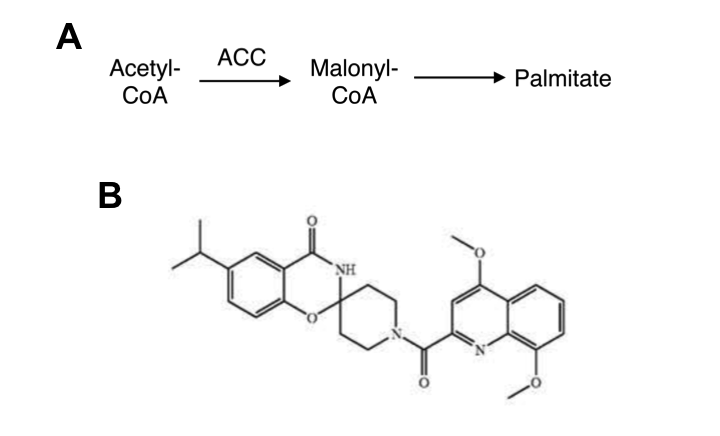

Supplement: S1 Fig — (A) ACC catalyzes the rate-limiting step of fatty acid synthesis by converting acetyl-CoA to malonyl-CoA. (B) Structure of novel ACC inhibitor, K1. (TIFF) [file pone.0156996.s001.tiff]

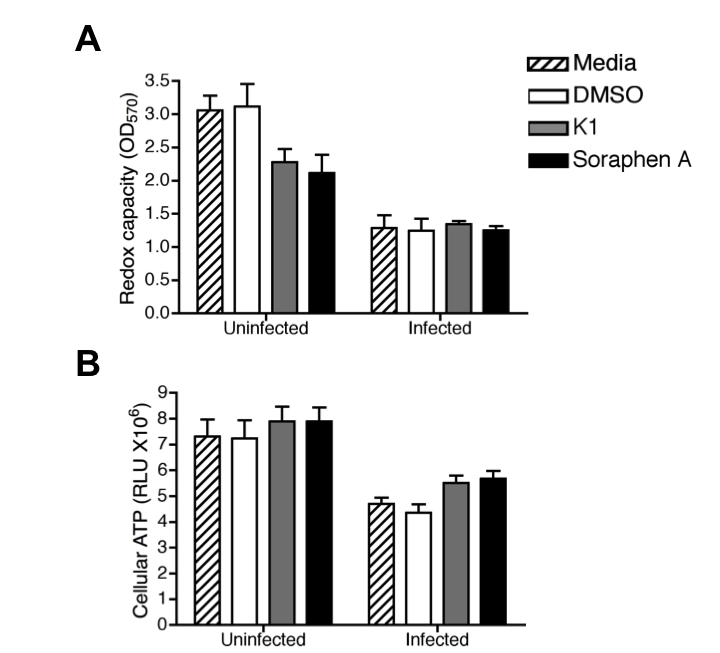

Supplement: S2 Fig — (A) Uninfected and infected Huh7.5.1 were grown in 96-well plates at 20,000 cells/well and treated with media, DMSO, 1 μM K1, or 100 nM soraphen A for 3 days. Effect on cell viability was determined by the MTT assay. Briefly, three hours before reading the absorbance, the cells were incubated at 37°C with 20 μL/well of 5 mg/mL of MTT (3-(4,5-dimethylthizaol-2-yl)-2,5-diphenyltetrazolium bromide). Precipitates were solubilized in isopropanol containing 4 mM HCl and 0.1% NP-40. Absorbance was read at 570 nm on a BioTek PowerWave XS. (B) Cells were grown as described in (A). Intracellular ATP content was quantified using the CellTiter-Glo® Luminescent Cell Viability Assay (Promega) according to the manufacturer’s instructions. Results are the mean ± SEM of 3 independent experiments. (TIFF) [file pone.0156996.s002.tiff]

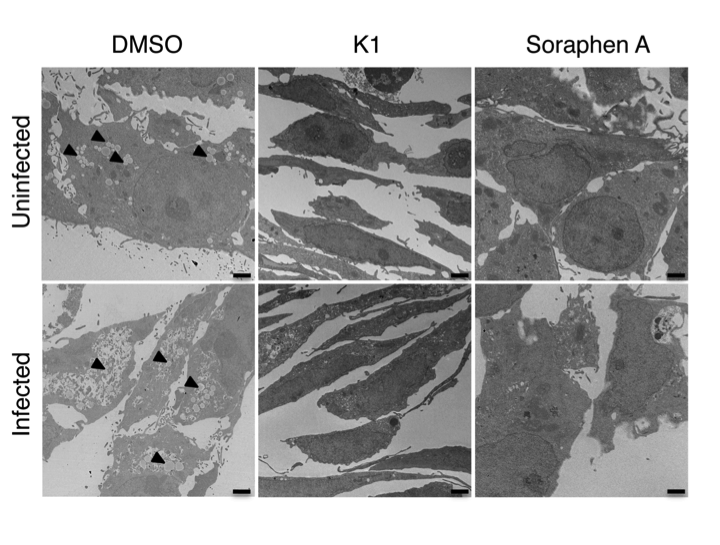

Supplement: S3 Fig — Infected Huh7.5.1 cells were treated with DMSO, K1, and Soraphen A for 3 days. Cell cultures were fixed in 4% paraformaldehyde/2.5% glutaraldehyde in PBS, post-fixed with 1% osmium tetroxide and potassium ferricyanide, dehydrated in ethanol, and embedded in Epon 812. Sections were cut on a Leica Ultracut UCT at a thickness of 60–80 nm and placed on 200 mesh copper grids for viewing in a JEOL 1010 transmission electron microscope. Images were obtained with a Hamamatsu ORCA-HR. Lipid droplets are indicated by the arrowheads. Scale bar is equivalent to 2 μm. Images are representative of 2–3 independent experiments. (TIFF) [file pone.0156996.s003.tiff]
