# Supplementary material for: Distinct Roles for Intracellular and Extracellular Lipids in Hepatitis C Virus Infection
Source: PLoS One. 2016 Jun 9;11(6):e0156996. doi: 10.1371/journal.pone.0156996 (PMC4900644; doi:10.1371/journal.pone.0156996)
Supplement: S1 Table — Infected Huh7.5.1 cells were treated with DMSO, 1 μM K1, or 100 nM soraphen A for 3 days. Indicated lipids were quantified by liquid chromatography tandem mass spectrometry (LC-ESI-MS/MS) and mean values were used to determine significant changes in K1 and soraphen A-treated cells compared to DMSO control. The column “p value” was determined by student’s t test and p<0.05 (bolded) was considered significant. The column “corrected p value” was adjusted for false discovery rate (FDR). Because FDR is more stringent than a t test, p<0.1 (bolded) was considered significant. Positive and negative values in the column “Log (fold change)” indicate an increase or decrease in the lipid, respectively. Results are the mean ± SEM of 3–4 independent experiments. (PDF) [file pone.0156996.s004.pdf]

S1 Table. Lipidomics of HCV-infected hepatocytes treated with ACC inhibitors.

Metabolite	Average±SD (relative abundance)			*p* value (*t* test)		Corrected *p* value (FDR adjusted)		Log (fold change)	
	DMSO	K1	Soraphen A	K1	Soraphen A	K1	Soraphen A	K1	Soraphen A
**Phosphatidylserine (PS)**									
16:0 16:0 PS	49.71±19.46	91.77±40.11	86.48±27.83	0.202178	0.133616	1.000000	1.000000	0.885	0.799
16:0 18:2 PS	402.35±94.31	330.43±199.23	419.17±153.36	0.607921	0.877173	1.000000	1.000000	-0.284	0.059
16:0 20:3 PS, 18:1 18:2 PS	729.21±181.59	432.57±281.26	656.49±296.05	0.202504	0.731185	1.000000	1.000000	-0.753	-0.152
16:0 20:4 PS	271.54±54.51	276.47±224.96	365.93±159.65	0.973495	0.416058	1.000000	1.000000	0.026	0.430
16:0 22:6 PS	248.20±87.76	249.86±185.07	268.11±95.09	0.989547	0.790307	1.000000	1.000000	0.010	0.111
18:0 18:1 PS	1003.80±275.01	542.50±292.46	488.46±177.84	0.097034	**0.030274**	1.000000	1.000000	-0.888	-1.039
18:0 20:2 PS	395.79±67.84	228.58±107.80	183.13±20.74	0.094620	**0.005188**	1.000000	0.513156	-0.792	-1.112
18:0 22:5 PS	1049.85±109.94	764.96±502.66	566.11±110.52	0.430640	**0.003291**	1.000000	0.342243	-0.457	-0.891
18:0 22:6 PS, 20:2 20:4 PS	3040.31±407.08	2053.23±1264.91	1555.42±239.81	0.307414	**0.001991**	1.000000	0.223047	-0.566	-0.967
18:1 18:1 PS, 18:0 18:2 PS	1329.13±264.22	640.52±361.86	670.65±197.64	0.056837	**0.013070**	1.000000	1.000000	-1.053	-0.987
18:1 20:4 PS, 18:0 20:5 PS	755.60±64.14	1047.70±706.97	1038.00±322.00	0.548803	0.266329	1.000000	1.000000	0.472	0.458
18:1 22:6 PS	411.16±59.44	795.01±435.24	676.60±115.15	0.265282	**0.039797**	1.000000	1.000000	0.951	0.719
**Phosphatidic acid (PA)**									
16:0 16:0 PA	19.56±13.97	17.07±10.50	14.45±0.89	0.798690	0.517809	1.000000	1.000000	-0.196	-0.437
16:0 18:1 PA	32.88±20.65	32.41±18.84	17.43±7.21	0.976040	0.239222	1.000000	1.000000	-0.021	-0.916
16:0 18:2 PA	17.29±7.25	8.96±0.18	8.62±4.64	0.184750	0.168513	1.000000	1.000000	-0.949	-1.004
16:0 20:3 PA	9.68±4.77	10.03±8.90	5.99±1.56	0.958634	0.463828	1.000000	1.000000	0.051	-0.693
16:0 20:4 PA	17.43±2.57	14.20±9.75	13.84±3.65	0.632087	0.290376	1.000000	1.000000	-0.296	-0.333
16:0 22:4 PA	8.36±5.11	2.69±4.43	1.69±0.86	0.179393	0.076512	1.000000	1.000000	-1.638	-2.309
16:0 22:6 PA	334.94±100.01	48.31±38.41	21.96±10.88	**0.006083**	**0.007678**	0.626550	0.744748	-2.794	-3.931
18:0 18:1 PA	22.12±5.84	16.13±10.68	16.55±2.05	0.446274	0.163634	1.000000	1.000000	-0.456	-0.418
18:0 18:2 PA	40.69±15.69	19.11±13.44	14.62±1.64	0.110047	**0.043915**	1.000000	1.000000	-1.090	-1.477
18:0 20:2 PA	42.95±26.46	10.41±4.09	27.78±18.24	0.163047	0.464960	1.000000	1.000000	-2.045	-0.629
18:0 20:4 PA	165.51±24.19	102.26±60.82	72.05±20.79	0.205899	**0.003088**	1.000000	0.324194	-0.695	-1.200
18:0 20:5 PA	8.33±4.58	1.82±0.88	2.58±0.33	0.126623	0.160536	1.000000	1.000000	-2.192	-1.693
18:0 22:5 PA	159.65±46.21	31.16±20.22	17.69±3.15	**0.006336**	**0.008332**	0.639964	0.791513	-2.357	-3.174
18:0 22:6 PA	578.15±231.02	99.73±45.11	76.33±1.64	**0.022753**	**0.022511**	1.000000	1.000000	-2.535	-2.921
18:1 18:2 PA	7.99±7.26	18.70±15.51	10.73±6.09	0.377838	0.704392	1.000000	1.000000	1.227	0.425
18:1 20:3 PA	9.87±5.44	19.11±10.47	15.95±7.23	0.263489	0.296354	1.000000	1.000000	0.952	0.692
18:1 20:4 PA	13.49±7.42	39.61±27.98	29.98±9.22	0.243901	0.066850	1.000000	1.000000	1.554	1.152
18:1 22:6 PA	1084.59±337.31	295.80±75.73	232.78±40.75	**0.015397**	**0.014032**	1.000000	1.000000	-1.874	-2.220

**Phosphatidylethanolamine (PE)**									
16:0 16:0 PE	0.39±0.13	0.16±0.07	0.12±0.03	**0.036451**	**0.025404**	1.000000	1.000000	-1.278	-1.645
16:0 18:0 PE	0.23±0.07	0.12±0.07	0.10±0.03	0.095342	**0.019541**	1.000000	1.000000	-0.969	-1.224
16:0 18:1 PE	2.46±0.32	1.45±0.78	1.18±0.31	0.142141	**0.001146**	1.000000	0.129508	-0.765	-1.061
16:0 18:2 PE	4.37±1.33	3.59±2.12	3.81±1.13	0.612640	0.542886	1.000000	1.000000	-0.283	-0.199
16:0 20:3 PE, 18:1 18:2 PE	3.93±1.42	3.08±1.94	3.82±1.46	0.561254	0.919111	1.000000	1.000000	-0.352	-0.040
16:0 20:4 PE	3.44±0.65	2.32±1.61	3.17±1.24	0.353082	0.723740	1.000000	1.000000	-0.567	-0.115
16:0 22:6 PE	3.23±0.76	2.66±1.86	3.32±1.23	0.653674	0.910816	1.000000	1.000000	-0.284	0.037
18:0 18:1 PE	0.86±0.17	0.68±0.40	0.47±0.09	0.526964	**0.012904**	1.000000	1.000000	-0.336	-0.860
18:1 18:1 PE, 18:0 18:2 PE	3.06±0.82	3.24±1.91	2.74±0.71	0.887555	0.582379	1.000000	1.000000	0.084	-0.157
18:1 20:3 PE, 18:0 20:4 PE	5.88±1.06	4.39±2.43	4.02±1.09	0.404677	0.050434	1.000000	1.000000	-0.421	-0.548
18:1 20:4 PE, 18:0 20:5 PE	4.25±0.98	4.93±3.46	5.51±2.05	0.771312	0.324530	1.000000	1.000000	0.213	0.375
**Phosphatidylcholine (PC)**									
16:0 16:0 PC	102.20±5.97	36.45±23.05	26.89±6.02	**0.033288**	**0.000002**	1.000000	**0.000250**	-1.488	-1.926
16:0 18:0 PC	31.58±0.90	10.76±7.41	7.00±2.08	**0.038406**	**0.000023**	1.000000	**0.002759**	-1.553	-2.173
16:0 18:1 PC	320.84±1.81	106.08±77.31	78.24±22.22	**0.040537**	**0.000195**	1.000000	**0.022981**	-1.597	-2.036
16:0 18:2 PC	196.41±17.84	137.13±93.06	116.79±28.58	0.384970	**0.005132**	1.000000	0.513156	-0.518	-0.750
16:0 20:3 PC	91.81±14.73	49.41±34.05	47.30±12.67	0.151887	**0.003982**	1.000000	0.410105	-0.894	-0.957
16:0 20:4 PC	63.14±4.93	40.45±28.02	47.70±15.93	0.294961	0.146204	1.000000	1.000000	-0.642	-0.405
16:0 22:6 PC	29.05±2.15	20.50±16.73	18.77±6.09	0.469672	**0.036769**	1.000000	1.000000	-0.503	-0.631
18:0 18:1 PC	45.74±2.43	17.58±13.00	11.24±3.37	0.060773	**0.000007**	1.000000	**0.000841**	-1.379	-2.024
18:1 18:1 PC	96.16±3.64	66.50±50.36	45.89±11.99	0.415027	**0.002140**	1.000000	0.233270	-0.532	-1.067
18:1 20:3 PC	36.23±3.79	27.98±21.70	22.33±4.98	0.578989	**0.005149**	1.000000	0.513156	-0.373	-0.698
18:1 20:4 PC	26.02±0.54	28.91±22.54	25.97±8.11	0.844969	0.990615	1.000000	1.000000	0.152	-0.003
**Diacylglycerol (DAG)**									
16:0 18:1 DAG	2.32±0.19	0.26±0.03	0.20±0.06	**0.000143**	**0.000054**	**0.017416**	**0.006436**	-3.142	-3.549
16:0 18:2 DAG	0.75±0.09	0.12±0.02	0.08±0.04	**0.000501**	**0.000215**	**0.058570**	**0.025206**	-2.642	-3.165
18:1 18:2 DAG	0.66±0.09	0.26±0.08	0.22±0.08	**0.002285**	**0.000401**	0.255877	**0.045726**	-1.351	-1.557
18:2 18:2 DAG	0.17±0.02	0.08±0.04	0.10±0.04	**0.029427**	**0.034102**	1.000000	1.000000	-1.116	-0.735
**Lysophosphatidic acid (LPA)**									
16:0 LPA	0.49±0.25	0.71±0.10	0.77±0.01	0.282166	0.203665	1.000000	1.000000	0.518	0.636
16:1 LPA	0.88±0.15	0.81±0.11	1.07±0.38	0.498958	0.490176	1.000000	1.000000	-0.123	0.274
18:0 LPA	8.59±0.76	5.74±0.89	6.38±1.29	**0.011156**	0.076047	1.000000	1.000000	-0.582	-0.429
18:1 LPA	0.88±0.34	1.94±0.26	2.10±0.27	**0.014968**	**0.009320**	1.000000	0.876099	1.142	1.262
18:2 LPA	0.44±0.19	0.52±0.09	0.65±0.15	0.532144	0.162008	1.000000	1.000000	0.225	0.565
20:4 LPA	1.37±0.38	2.02±0.38	1.99±0.35	0.081698	0.078755	1.000000	1.000000	0.559	0.537

Sphingomyelin (SM)									
SM 13:0	6.70±0.81	2.20±0.51	3.86±1.13	**0.000301**	**0.008078**	**0.035866**	0.775466	-1.608	-0.794
SM 13:1	1.15±0.14	0.27±0.21	0.48±0.32	**0.006176**	**0.016830**	0.629968	1.000000	-2.114	-1.264
SM 14:0	12.08±1.47	14.20±0.75	11.20±2.33	0.059186	0.547937	1.000000	1.000000	0.233	-0.110
SM 14:1	0.68±0.26	1.19±0.17	0.85±0.37	**0.025966**	0.483112	1.000000	1.000000	0.806	0.324
SM 15:0	17.79±4.72	16.71±1.63	13.46±2.53	0.694089	0.171895	1.000000	1.000000	-0.090	-0.403
SM 15:1	0.52±0.32	0.72±0.21	1.06±0.67	0.364432	0.215315	1.000000	1.000000	0.472	1.027
SM 16:0	284.22±61.23	210.69±41.84	177.57±27.60	0.118086	**0.031748**	1.000000	1.000000	-0.432	-0.679
SM 16:1	32.30±7.14	27.34±1.44	31.55±6.38	0.260248	0.879598	1.000000	1.000000	-0.241	-0.034
SM 17:0	11.05±1.86	6.38±1.41	7.03±0.74	**0.012996**	**0.016602**	1.000000	1.000000	-0.793	-0.653
SM 17:1	2.74±0.76	2.74±0.06	2.96±0.40	0.996469	0.639693	1.000000	1.000000	-0.001	0.109
SM 18:0	17.18±2.23	6.42±1.68	9.74±0.81	**0.000777**	**0.004029**	**0.089300**	0.410988	-1.421	-0.819
SM 18:1	7.78±1.01	4.43±0.48	7.49±1.86	**0.003066**	0.792757	0.334196	1.000000	-0.812	-0.056
SM 19:0	1.58±0.20	0.78±0.36	1.78±0.37	**0.039957**	0.395339	1.000000	1.000000	-1.031	0.170
SM 19:1	0.47±0.20	0.24±0.03	0.63±0.15	0.105426	0.251236	1.000000	1.000000	-0.966	0.427
SM 20:0	7.52±1.23	3.17±1.24	8.81±0.42	**0.007897**	0.123190	0.789704	1.000000	-1.247	0.229
SM 20:1	2.76±0.25	1.17±0.09	3.48±0.47	**0.000310**	**0.046283**	**0.036544**	1.000000	-1.232	0.336
SM 21:0	1.87±0.37	0.99±0.43	2.77±0.38	**0.047068**	**0.014477**	1.000000	1.000000	-0.918	0.565
SM 21:1	0.73±0.14	0.47±0.15	1.00±0.21	0.073739	0.080126	1.000000	1.000000	-0.643	0.454
SM 22:0	10.66±1.14	5.03±2.11	13.44±1.83	**0.026818**	**0.049042**	1.000000	1.000000	-1.083	0.334
SM 22:1	9.09±0.98	4.99±1.17	12.89±1.11	**0.008351**	**0.002260**	0.826705	0.244048	-0.865	0.503
SM 22:2	0.71±0.35	0.47±0.13	1.41±0.40	0.268133	**0.039734**	1.000000	1.000000	-0.607	0.986
SM 23:0	3.30±0.30	1.75±0.78	3.79±0.98	0.063349	0.396060	1.000000	1.000000	-0.915	0.201
SM 23:1	4.22±0.99	2.30±0.74	6.72±0.88	**0.032752**	**0.009610**	1.000000	0.893712	-0.875	0.671
SM 24:0	9.26±1.55	5.46±1.77	7.54±0.83	**0.040821**	0.112845	1.000000	1.000000	-0.761	-0.296
SM 24:1	22.13±1.57	11.95±2.91	23.34±2.44	**0.013193**	0.443613	1.000000	1.000000	-0.889	0.076
SM 24:2	6.69±0.56	5.22±0.52	8.69±1.14	**0.017856**	**0.029834**	1.000000	1.000000	-0.358	0.379
SM 24:3	0.56±0.14	0.59±0.12	1.02±0.11	0.777744	**0.002429**	1.000000	0.259912	0.074	0.865
SM 25:0	0.65±0.09	0.46±0.14	0.67±0.15	0.120852	0.885684	1.000000	1.000000	-0.493	0.029
SM 25:1	1.28±0.11	0.96±0.16	1.43±0.48	**0.049447**	0.579077	1.000000	1.000000	-0.404	0.160
SM 26:0	0.21±0.11	0.14±0.05	0.15±0.06	0.381259	0.426109	1.000000	1.000000	-0.576	-0.502
SM 26:1	0.60±0.14	0.40±0.07	0.52±0.11	0.113538	0.449407	1.000000	1.000000	-0.601	-0.214
SM 26:2	0.35±0.18	0.42±0.04	0.49±0.24	0.509427	0.389852	1.000000	1.000000	0.262	0.489

**Glucosylceramide (GlcCer)**									
GlcCer 16:0	166.12±39.31	7.56±2.69	154.12±70.17	**0.003839**	0.778191	0.406884	1.000000	-4.458	-0.108
GlcCer 18:0	15.22±3.42	0.71±0.27	15.39±8.69	**0.003272**	0.971997	0.353400	1.000000	-4.426	0.016
GlcCer 20:0	3.71±0.77	0.16±0.03	3.36±2.51	**0.002681**	0.801857	0.297637	1.000000	-4.575	-0.145
GlcCer 21:1	0.69±0.09	0.04±0.01	0.49±0.18	**0.000675**	0.105091	**0.078310**	1.000000	-4.245	-0.500
GlcCer 21:2	3.41±1.28	0.13±0.02	1.69±0.85	**0.014272**	0.073064	1.000000	1.000000	-4.689	-1.011
GlcCer 22:0	3.02±1.15	0.12±0.02	2.07±1.27	**0.015058**	0.311919	1.000000	1.000000	-4.696	-0.545
GlcCer 22:1	0.55±0.18	0.04±0.00	0.40±0.25	**0.010844**	0.370702	1.000000	1.000000	-3.765	-0.457
GlcCer 23:0	1.03±0.25	0.05±0.02	0.70±0.46	**0.004494**	0.277920	0.471912	1.000000	-4.269	-0.549
GlcCer 23:1	0.78±0.21	0.04±0.01	0.48±0.28	**0.005393**	0.133275	0.560847	1.000000	-4.147	-0.719
GlcCer 24:0	3.12±1.01	0.14±0.02	1.38±0.49	**0.009664**	**0.032506**	0.937428	1.000000	-4.530	-1.177
GlcCer 24:1	2.32±0.89	0.12±0.01	1.23±0.45	**0.015819**	0.088016	1.000000	1.000000	-4.305	-0.913
**Dihydroceramide (DCer)**									
DCer 16:0	33.97±5.41	28.47±7.47	23.20±13.84	0.348048	0.222329	1.000000	1.000000	-0.255	-0.550
DCer 18:0	3.74±0.88	3.46±1.19	3.16±0.66	0.744985	0.331663	1.000000	1.000000	-0.115	-0.246
DCer 20:0	5.64±1.58	4.56±0.95	2.99±2.11	0.315644	0.095184	1.000000	1.000000	-0.306	-0.916
DCer 22:0	3.58±0.92	3.02±1.39	2.27±0.45	0.588981	0.058173	1.000000	1.000000	-0.242	-0.657
DCer 24:0	5.00±0.70	4.86±0.62	4.27±1.97	0.787436	0.524919	1.000000	1.000000	-0.042	-0.228
DCer 24:1	7.81±1.59	6.95±2.18	4.72±2.39	0.600409	0.082206	1.000000	1.000000	-0.167	-0.725
**Ceramide (Cer)**									
Cer 16:0	270.73±39.69	119.80±7.65	133.62±17.98	**0.003651**	**0.002803**	0.390629	0.297066	-1.176	-1.019
Cer 18:0	35.05±4.06	10.94±0.67	15.35±3.16	**0.000969**	**0.000344**	0.110420	**0.039885**	-1.680	-1.191
Cer 18:1	6.08±1.16	2.78±0.22	4.74±1.30	**0.009031**	0.176240	0.885021	1.000000	-1.128	-0.359
Cer 20:0	24.67±6.52	7.59±1.32	17.34±8.32	**0.011315**	0.217307	1.000000	1.000000	-1.700	-0.509
Cer 20:1	2.67±0.89	1.03±0.44	1.98±0.60	**0.027171**	0.247543	1.000000	1.000000	-1.379	-0.437
Cer 20:2	4.00±0.95	1.28±0.09	3.86±2.03	**0.009895**	0.910702	0.949943	1.000000	-1.647	-0.049
Cer 22:0	33.90±4.65	12.37±3.69	20.34±3.48	**0.001082**	**0.004177**	0.122302	0.421879	-1.455	-0.737
Cer 22:1	4.39±0.23	2.00±0.27	3.16±0.36	**0.000247**	**0.002038**	**0.029835**	0.226179	-1.136	-0.476
Cer 23:0	31.70±11.78	10.85±3.71	29.19±10.96	**0.032164**	0.766056	1.000000	1.000000	-1.547	-0.119
Cer 24:0	71.75±8.25	20.60±3.62	26.81±4.21	**0.000251**	**0.000362**	**0.030143**	**0.041607**	-1.800	-1.420
Cer 24:1	76.85±10.45	40.71±7.17	41.53±7.54	**0.002901**	**0.002095**	0.319120	0.230429	-0.916	-0.888

Infected Huh7.5.1 cells were treated with DMSO, 1 mM K1, or 100 nM soraphen A for 3 days. Indicated lipids were quantified by liquid chromatography tandem mass spectrometry (LC-ESI-MS/MS) and mean values were used to determine significant changes in K1 and soraphen A-treated cells compared to DMSO control. The column “p value” was determined by student’s t test and $p<0.05$ (bolded) was considered significant. The column “corrected p value” was adjusted for false discovery rate (FDR). Because FDR is more stringent than a t test, $p<0.1$ (bolded) was considered significant. Positive and negative values in the column “Log (fold change)” indicate an increase or decrease in the lipid, respectively. Results are the mean ± SEM of 3-4 independent experiments.
